# USRCAT: real-time ultrafast shape recognition with pharmacophoric constraints

**DOI:** 10.1186/1758-2946-4-27

**Published:** 2012-11-06

**Authors:** Adrian M Schreyer, Tom Blundell

**Affiliations:** 1 Department of Biochemistry, University of Cambridge, 80 Tennis Court Road, CB2 1GA, Cambridge, United Kingdom

**Keywords:** Virtual screening, Ultrafast shape recognition

## Abstract

**Background:**

Ligand-based virtual screening using molecular shape is an important tool for researchers who wish to find novel chemical scaffolds in compound libraries. The Ultrafast Shape Recognition (USR) algorithm is capable of screening millions of compounds and is therefore suitable for usage in a web service. The algorithm however is agnostic of atom types and cannot discriminate compounds with similar shape but distinct pharmacophoric features. To solve this problem, an extension of USR called USRCAT, has been developed that includes pharmacophoric information whilst retaining the performance benefits of the original method.

**Results:**

The USRCAT extension is shown to outperform the traditional USR method in a retrospective virtual screening benchmark. Also, a relational database implementation is described that is capable of screening a million conformers in milliseconds and allows the inclusion of complex query parameters.

**Conclusions:**

USRCAT provides a solution to the lack of atom type information in the USR algorithm. Researchers, particularly those with only limited resources, who wish to use ligand-based virtual screening in order to discover new hits, will benefit the most. Online chemical databases that offer a shape-based similarity method might also find advantage in using USRCAT due to its accuracy and performance. The source code is freely available and can easily be modified to fit specific needs.

## Background

Three-dimensional shape is a fundamental molecular property that can be directly observed in electron density maps obtained through X-ray crystallography. Given the assumption that shape complementarity is a prerequisite for binding between a ligand and its receptor, it follows that active molecules with similar shapes could be active against the same targets. Shape-based *virtual screening* has therefore become a popular method to find a set of molecules that resemble a reference structure known to be active against the protein target of interest. Ligand-based shape similarity has a number of inherent advantages; for instance protein structures are not needed, so that virtual screens can be carried out with any biological target as long as there is at least one reference active molecule available. Furthermore, shape-based algorithms are capable of retrieving compounds that do not share any topological similarity. This ability, called *scaffold hopping*, is an important characteristic of these methods and arguably the reason for their popularity. Shape-based virtual screening thereby avoids problems with already patented scaffolds (that might be used as templates for searching). Most importantly, it can retrieve several distinct scaffolds as top-ranked hits that can be pursued individually.

Shape comparison algorithms can be broadly divided into two different groups: *alignment-based* and *moment-based* methods. Alignment-based methods relying on molecular superposition retain almost all of the shape information of a molecule but do not encode shape in a numerical form. Although they are computationally expensive, they enable precise geometric comparison of surface features such as polarity and hydrophobicity as well as chirality at the same time. Moment-based methods, on the other hand, attempt to compress the shape information through various approximations into a rotationally and translationally invariant, numerical form. Time-consuming molecular alignments can be omitted (in most cases) and since numerical representations can be conveniently stored in a database, the screening of very large conformer databases becomes feasible. Such moment-based methods do not usually retain any of the pharmacophoric information of the encoded molecule; therefore additional measures are often necessary to ensure that retrieved molecules have chemical properties that are similar to the reference geometry. A substantial amount of research has been carried out in this field in recent years and a few algorithms as well as case studies have been published [1]. Furthermore, ligand-based virtual screening relying on shape alone was found to deliver performances comparable to protein-ligand docking [2,3].

### Ultrafast shape recognition

Ultrafast Shape Recognition (USR) [4,5] is a moment-based virtual screening method that uses the relative position of bonded atoms to describe molecular shape. The shape is characterised by a set of distributions that are created by measuring the distance between the atoms and four reference points. The final shape is encoded as the first three statistical moments of these distance distributions resulting in a vector with 12 elements (USR moments) that is unique for a set of coordinates. The distance distributions are completely independent of molecular orientation (rotation) and position (translation). Hence, USR moments only have to be calculated once for each molecule and their comparison to calculate shape similarity does therefore not require superposition unlike most other shape-based methods. The compact representation of only 12 values and an extremely fast similarity calculation (variant of the Manhattan distance) makes the USR algorithm very suitable for usage in a database system in the form of a native implementation. USR has been used successfully in several prospective virtual screening campaigns where *in silico* hits were confirmed experimentally as biologically active [6,7].

There also have been a few extensions of USR developed in recent years to augment the method with descriptors to address the lack of discrimination between enantiomers as well as between compounds having similar shape but different atomic properties. One of the first was a hybrid approach between USR and MACCS key descriptors to add structural properties [8]. Subsequent extensions tried to tackle the lack of discrimination between chiral compounds [9,10]. The latest development has been ElectroShape, a variant of USR that encodes electrostatics and optionally liphophilicity through additional dimensions and centroids [11,12].

### The CREDO structural interactomics database

The CREDO structural interactomics database [13] contains the interatomic interactions between all macro- and small molecules found in three-dimensional structures stored in the Protein Data Bank (PDB) [14]. It also contains the chemical components that constitute the residues and ligands in PDB structures. An important entry process into the database is to find ligands that are similar to a reference compound in order to find potential protein targets, identify secondary off-target effects or to analyse the structural interactions of the retrieved ligands with their protein binding sites. Cheminformatics extensions, often called cartridges, are available for the most commonly used relational database management systems (Oracle, MySQL, and PostgreSQL) either as commercial software or freely-available open source projects [15-18]. All of these extensions support traditional graph-based query methods such as substructure search, pattern match or topological similarity that can be used to retrieve compounds similar to a reference molecule. The CREDO database uses the PostgreSQL as its underlying relational database management system (RDBMS) and implements the open-source RDKit [18] as well as an extension using the OpenEye toolkits ( http://www.eyesopen.com) for this purpose.

Missing so far has been an entry process that allows users to retrieve ligands that have shapes similar to a query molecule and consequently would potentially allow inferring knowledge (such as biological targets, interactions) from the ligand onto the query. The usage of shape-based methods as an entry process has not been feasible in the past due to either storage issues or the lack of query performance. In reality, only moment-based methods are suitable for implementation into a database system supporting a public web site and RESTful [19] web services. The more computationally demanding molecular superposition-based approach is far too computationally expensive (although the usage of ROCS in CREDO is possible through a database extension). The development and implementation of the USR has ensured a shape-based method with possible millisecond response time due to its computational efficiency.

### Ultrafast shape recognition with CREDO atom types

The *Ultrafast Shape Recognition with CREDO Atom Types* (USRCAT) extension of the USR algorithm was developed as part of the CREDO database and web service to enable users to find chemical components (and their ligands) in the Protein Data Bank (PDB) that are similar to a reference molecule. However, chemical components in the PDB are extremely diverse, ranging from natural products, drug-like molecules to solvents, redox agents, ion chelators and more exotic structures such as metal clusters. The traditional USR algorithm is agnostic of atom types and cannot discriminate between structures with potentially similar shapes, independent of their substructures or functional groups. In a typical virtual screening campaign, the compound libraries are rigorously filtered to contain only drug-like molecules within a narrow range of chemical properties. Here, the lack of pharmacophoric discrimination is likely to have less of an impact on the actual performance. With the highly heterogeneous chemical components in the CREDO database however, the lack of discrimination between atom types becomes apparent and the USR approach is found to be insufficient - top-ranked screening hits often contain inappropriate compounds for the given query molecule such as carbohydrates and solvents that are frequently found amongst crystal structures. One solution for this problem has been found using Ultrafast Shape Recognition with Atom Types (UFSRAT) [20,21], an extension of USR that encodes pharmacophoric features with atom types. The concept of UFSRAT is simple, meaning the advantages of USR, namely compact storage and very fast screening, are retained. While the initial results of using UFSRAT with the chemical components in CREDO were promising, the extension could be further improved, eventually leading to the development of USRCAT.

## Results and discussion

**Figure 1:**
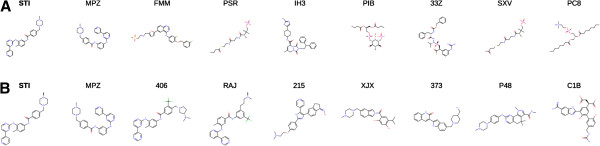
**Comparison of the top ten hits of a USR and USRCAT search with Imatinib used as query against chemical components found in the Protein Data Bank (PDB). A**, Top ten hits retrieved with the traditional USR method. **B**, Top ten hits retrieved by a USRCAT search with all pharmacophore weigts and probe radius set to 1.0.

The advantages of the USRCAT method can be seen easily in a simple screen of a heterogeneous set of molecules. Figure 1 shows the top ten results of a USR/USRCAT search with Imatinib (HET-ID:STI) against the conformers of all chemical components in the PDB (July 2012, 14,556 entries). Figure 1A shows the structures of the top hits with traditional USR whereas Figure 1B shows the results from USRCAT with all pharmacophore weights and the radius of the bounding box (probe radius) set to 1.0 (see the methods section for an explanation of pharmacophore weights and probe radius).

The comparison based on a heterogeneous data set shows the benefits of the USRCAT extension immediately. The traditional USR method is capable of retrieving highly-similar (HET-ID:MPZ, HET-ID:FMM) hits at the top of the returned results but struggles with compounds that appear to have similar atomic coordinate distributions but are completely unrelated in terms of their pharmacophoric profiles (HET-ID:PSR, HET-ID:PIB). Aromaticity was implemented in USRCAT as a pharmacophoric subset (not in UFSRAT) because USR was unable to discriminate between long, chain-like molecules such as certain heteropeptides and long alkyl chains in particular. The extra descriptor solved this problem in the test data set (Figure 1).

There is an important caveat however that was not addressed in the UFSRAT implementation, namely what happens if there are not enough atoms in a subset to create a distribution and/or to calculate the first three statistical moments. UFSRAT treats the atom subsets as completely independent, i.e. the four reference points are calculated for each set of atoms individually and the statistical parameters subsequently derived from the distance distributions of those points. This is problematic because either the parameters cannot be calculated at all or the underlying distance distributions will not be meaningful. In the context of virtual screening this is very likely to affect the performance of the method because some pharmacophoric features are rarer than others, particularly in compound libraries optimised for drug-likeness. Hydrogen bond donors for example are normally scarce – more than five would violate Lipinski’s rule of five. This is the case in the set of bioactive compounds from the DUD-E database that were used for benchmarking purposes as they contained a mean of 2.18 hydrogen bond donors. To address this issue, USRCAT re-uses the four reference points from the entire atom distribution. This has the advantage that the moments derived from a given subset with the very same atoms are more discriminatory and, most importantly, they provide an encoding of the location of the pharmacophoric features with respect to the overall shape of a molecule.

### Retrospective virtual screening benchmark with DUD-E

**Figure 2:**
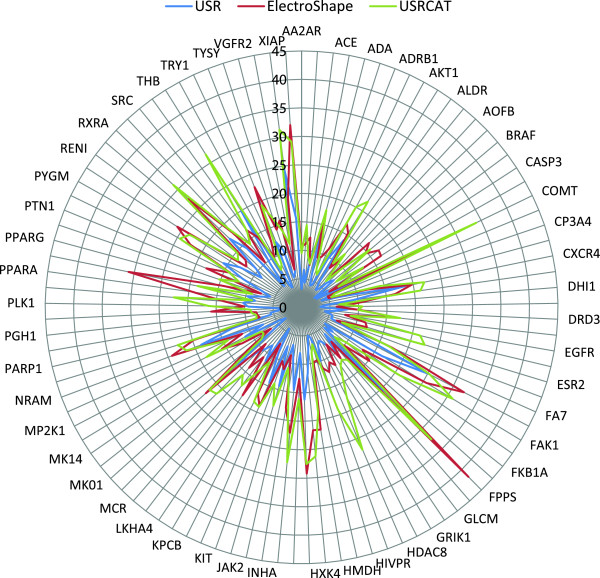
**Comparison of enrichment factors achieved by the USR, Electroshape and USRCAT methods.** The radar plot shows the enrichment factors at the 0.25% level achieved by the USR (blue), Electroshape (red) and USRCAT (green) methods in the restrospective virtual screening benchmark using the DUD-E dataset. No index was used and all pharmacophore weights were set to 1.0 for the USRCAT method.

A retrospective virtual screening benchmark was performed to establish whether USRCAT can improve the performance of the traditional USR method and be at least competitive with the ElectroShape method that also includes pharmacophoric information in the form of electrostatics and lipophilicity. In this case, the Directory of Useful Decoys, Enhanced (DUD-E) [22], a benchmarking set for molecular docking that includes diverse targets such as GPCRs and ion channels, was used. DUD-E contains 102 targets with clustered ligands from ChEMBL [23] and an average of 50 decoys for each active with matching physicochemical properties from ZINC. An enrichment factor was used as the measure to compare the two methods because it emphasises the top-ranked results and is a realistic estimator of the performance in a real-world (prospective) virtual screening campaign – where only a certain number of top-ranked hits are considered for further testing [24]. The obtained enrichment factors averaged over all DUD-E targets for each method can be seen in Table 1. As can be seen, USRCAT was able to outperform the traditional USR method at every enrichment factor level that was considered, achieving almost twice the enrichment at the most important 0.25% level. For some targets, the performance boost was even more dramatic (e.g. *COMT* +21.48 EF 0.25%, *HDAC8* +19.82, *ESR2* +16.87). The enrichment factors (EF) at the 0.25% level for each target in DUD-E are displayed in Figure 2. Interestingly, *ACES* (Acetylcholinesterase) was the only target where the performance at EF 0.25% did not improve with both USRCAT (−1.14 EF 0.25%) and ElectroShape (−1.30) compared to USR. A table containing the achieved enrichment factors for all targets and analysed levels is available with the online version of this paper ( Additional file 1). USRCAT also achieved slightly higher enrichment on average than ElectroShape in this benchmark but the results varied strongly between DUD-E targets. For example, USRCAT was significantly better with some targets (*THB* +17.15 EF 0.25%, *HDAC8* +14.84, *MET* +12.29) whereas ElectroShape achieved higher enrichment with others (*PPARD* +12.37 EF0.25%, *PPARA* +10.4). USRCAT ultimately performed better with 64 of the DUD-E targets and ElectroShape consequently with the remaining 37.

**Table 1:** Average enrichment factors obtained in a retrospective virtual screen of the DUD-E database (no pooling)

**Method**	**Enrichment Factors**		
	**1.0%**	**0.5%**	**0.25%**
USR	5.00	6.71	8.84
ElectroShape	8.40	11.27	14.48
USRCAT	8.62	11.99	15.64
Circular FP	32.14	42.54	49.72
Path FP	24.50	35.14	44.27
Tree FP	19.28	28.98	38.59
MACCS166	20.90	28.85	36.60

Interestingly, there is a correlation (ElectroShape: r=0.72, MACCS166: r=0.68, tree: r=0.67, path: r=0.61, circular: r=0.51) between the enrichment factors achieved by USRCAT and the other methods used in this benchmark. This relationship indicates that virtual screening performance strongly depends on the DUD-E target sets. A closer look on the obtained enrichment factors for each target reveals that all methods performed worst against the *CP3A4* and *CP2C9* targets (both cytochrome P450s). The same is true for protein-ligand docking: the DUD-E web site ( http://dude.docking.org) shows that this method performed poorly against these targets as well. On the other side of the spectrum, all used methods achieved their best results against *FPPS* and *COMT*.

Another important question is whether using all conformers of an active query molecule would improve performance further compared to just using the Lowest Energy Conformer (LEC). The implementation of the benchmark data set in a relational database made this easy to analyse: instead of using only a single query, all the rows containing the USRCAT moments of the query were used in a cross-join (an all-by-all comparison) and the highest scoring combination of both query and target conformer was taken. However, this did not improve the screening performance at all, possibly because decoys are more likely to be similar to higher energy conformers of an active query molecule. A benchmark was also performed with LECs only (LEC of active query molecules and LECs in the target set). Surprisingly, that benchmark achieved exactly the same enrichment factors as the one against all the target conformers. Closer inspection revealed that in all the checked instances, the LEC of a retrieved active had the highest similarity. As a side note, conformers generated by OpenEye’s Omega toolkit can be assigned numbers to identify them. Since conformers are also sorted by increasing strain energy, conformer number 0 of a molecule is the LEC. Importantly, once the LECs were excluded on the target side (conformer number > 0) then the pattern of retrieved conformer serial numbers becomes far more diverse and the number of retrieved actives is significantly reduced too. The highest enrichment factors in this study were only achieved if the LEC of an active was used as a query and if the LECs were included in the target set. These observations however only reflect the used data set (DUD-E) in combination with the used conformer generation tool (Omega TK) and cannot be generalised.

During this benchmark it became apparent that the virtual screening performance of all the tested variants of USR is highly dependent on the query molecule and the compound library that is screened. Decoys in DUD-E were chosen to resemble the active ligands in terms of their physicochemical properties whilst being *topologically dissimilar to minimize the likelihood of actual binding*[22]. In fact, rigorous filtering was performed to ensure that no “warheads” remain in the decoy sets. Hence, shape-based algorithms have a significant disadvantage in this benchmark compared to the methods relying on topological similarity. Thus, the very high enrichment factors achieved by the topological similarity methods can be explained with the design of the DUD-E database, which was intended to be used as a tool to benchmark protein-ligand docking methods and not topological fingerprints. Moreover, active ligands are clustered by their Bemis–Murcko scaffolds to increase their diversity. As a result, chemicophysical properties of the actives within a target set, such as molecular weight, very significantly ( [22], Additional file 1: Table S3). The huge variation in molecular size of the active ligands in the target sets was detrimental to the virtual screening performance of USR/ElectroShape/USRCAT. The USR algorithm is a global measure and thus not effective at detecting similarity between molecules of very different sizes. The average standard deviation for the heavy atom count of the actives in the target sets was 5.3 and the average difference in heavy atom count between the largest and smallest active ligand in a target set was 26.5.

Consequently, the performance of the USRCAT method could be increased in several ways. First, the size of the molecules that are used as queries should be well within the range of the molecular sizes in the compound library that is to be screened. Second, the pharmacophoric weights should be adjusted for a particular target and the properties of the protein-ligand binding site (if known) – it is very likely that depending on the target some properties might be more important than others. Third, the pharmacophore subsets of the query molecule could be restricted to contain only atoms that are known to make interactions with the protein binding site of the biological target (this requires a reference structure of the protein-ligand complex). It should be remembered that some of the functional groups of a drug do not contribute to binding affinity but are only added during lead development to improve other important properties such as bioavailability. Excluding these atoms from the pharmacophore subset could therefore reduce the false positive rate.

### Execution time analysis

**Figure 3:**
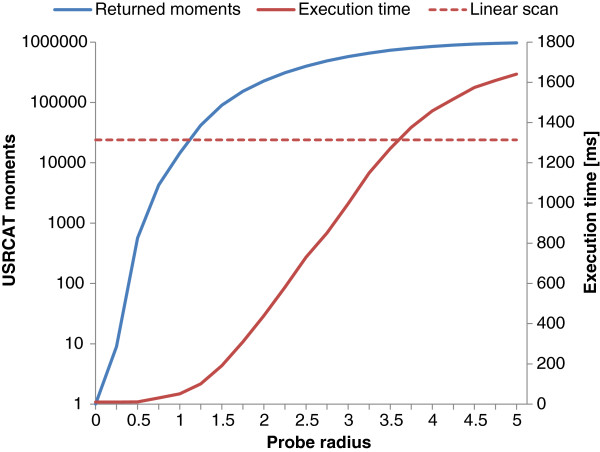
**Impact of the probe radius on USRCAT query performance.** The blue chart line shows the number of USRCAT moments that were included in a bounding box created by enlarging the USR space cube of the reference ligand by the given radius. The solid red line shows the time taken (in milliseconds) to execute the full query (incl. sorting) for the given probe radius. The execution time of a linear table scan is displayed with the red dashed line. The DUD-E target with the largest number of active and decoys, *FNTA*, was used for this performance analysis test.

Speed is a crucial aspect of virtual screening as well as modern web services. A SQL query with *pgopeneye* using a linear scan of all 1,431,121 circular fingerprints (with 2048 bits) took less than 900 ms to calculate each Tanimoto index, order the result and return the top ten hits. This result corresponds to a screening performance of over 1.6M fingerprints per second. Alternatively, using the Generalized Search Tree (GIST) index reduces time to less than 500 ms. The screening performance of the USRCAT implementation depends on the selected radius for the bounding box created by the USR space cube (in case the cube GIST index is used). As an example, the DUD-E target with the largest number of actives and decoys, *FNTA*, contained 1,021,602 conformers with USRCAT moments. A linear scan including sorting, i.e. calculating the similarity to all conformers in the corresponding table, took 1314 ms (the best of three trials). The execution time of USRCAT queries using the cube GIST index strongly correlates with the selected probe radius: the larger the radius, the more USR moments are included in the query bounding box. The relationship between probe radius and execution time can be seen below in Figure 3.

A bounding box enlarged by a probe radius of zero only contains the USRCAT moments that are used as reference for a query. The cube GIST index appears to be only beneficial if the probe radius is below 3.5, otherwise the index will contain too many pages (files on disk) and a linear table scan will be faster. Also, bounding boxes enlarged by probe radii larger than 3.0 only include marginally more USR moments. It should also be noted here that the database performance depends on the underlying physical storage – the use of solid state media instead of spinning disks would further improve speed considerably.

### Impact of the probe radius on the DUD-E benchmark enrichment factor

**Figure 4:**
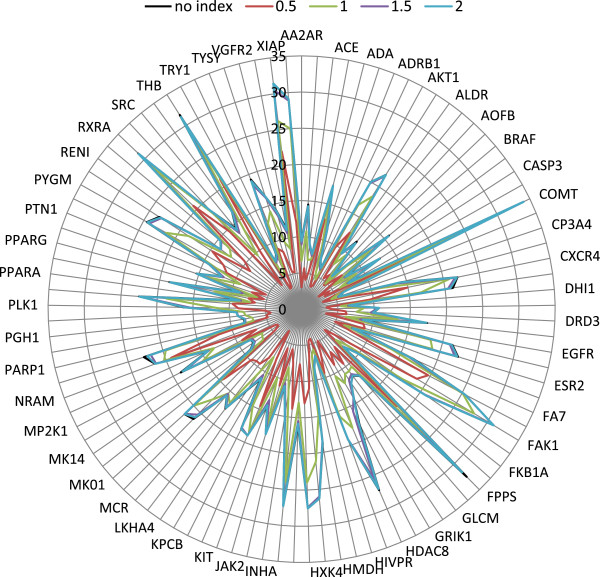
**Impact of the probe radius on the DUD-E benchmark enrichment factors.** The radar plot shows the Enrichment Factors (EF) at the 0.25% level for each target in DUD-E obtained through a retrospective virtual screen using USRCAT with different probe radii. All four pharmacophore weights were set to 1.0 in all screens.

It was also necessary to analyse the impact of the selected probe radius, by which the bounding box is enlarged, on the benchmark enrichment factors. The use of the cube GIST index limits the number of returned rows that are included for similarity calculation, which means that not all USRCAT moments are actually screened. The bounding box created by the probe radius only depends on the first 12 USRCAT moments that reflect the shape characteristics of all atoms. This means that the smaller the probe radius, the closer the result from USRCAT converges towards classical USR. In contrast, a larger probe radius would include more of the molecules with less global USR similarity, which in turn would increase the dependence of the four pharmacophore moments on the calculated similarities. Figure 4 shows the 0.25% enrichment factor for each target in DUD-E and for a sensible range of possible probe radii (from 0.5 to 2.0).

As can be seen in the radar plot, a very small probe radius used for screening affected the virtual screening outcome in this benchmark significantly. As a reference, a screen using linear scans and no index achieves an EF 0.25% of 15.64 and a probe radius of 0.5 achieves an EF 0.25% of 8.27 (which is also very close to the value of 8.84 for USR). The performance gap closes down with increasing probe radius however: with radius 1.0 the EF 0.25% increases to 12.9 and starts to converge at larger values (1.5: 15.28, 2.0: 15.56, 2.5: 15.62). As a result, the probe radius of 1.5 would have been optimal for the compounds that comprise the DUD-E dataset because it achieves a 7x reduction in execution time compared to a linear scan whilst the impact on screening performance is only minimal. In other words, using this probe radius would have corresponded to an effective screening speed of more than 5.3M conformers per second. The probe radii used in this benchmark however were increased to optimise virtual screening performance (similar to the large scaling factors used in the ElectroShape benchmark). Given the large variations in the size of the active ligands in DUD-E, a smaller probe radius would be sufficient to effectively screen a database of compounds having a narrower distribution of molecular size.

### Scaffold hopping potential

A highly desirable outcome of ligand-based virtual screening using shape similarity is the ability to find active compounds that do not possess any significant topological similarity to a given reference molecule. High scaffold hopping potential would mean in this context that the USRCAT extension is capable of retrieving actives with low Tanimoto similarity to query molecules that would not be returned by using topological (fingerprint) similarity. The retrieved active hits (at the 0.25% EF level) together with the calculated Tanimoto similarity (using circular fingerprints) were stored in a separate table for each active ligand used as a query in the enrichment factor benchmark. The data in this table were used to calculate the minimum, maximum and average number of actives for each target in DUD-E that were retrieved for the active query molecules with USRCAT but not with topological similarity. The results are derived from the virtual screen with USRCAT that achieved the best enrichment factors (all moments set to 1.0, no index) and can be seen in Table 2. Active query molecules were only included in this analysis if both methods were able to retrieve hits for them. As an example, for target *CDK2* each query retrieved at least one hit that could not be retrieved by topological similarity and at least one query was capable of retrieving 25 hits not found otherwise. Each active query molecule retrieved on average 6.48 hits not returned by screening with the topological (circular) fingerprints. Although screening by topological similarity generally performed better in this benchmark due to the intrinsic properties of the DUD-E database, the USRCAT extension was able to retrieve hits that could not be retrieved otherwise for almost all active query molecules.

**Table 2:** A summary of the number of hits that were retrieved by USRCAT but not by using topological similarity as the criterion

**Target**	**Min**	**Max**	**Avg**	**Target**	**Min**	**Max**	**Avg**	**Target**	**Min**	**Max**	**Avg**
*AA2AR*	1	26	5.92	*FAK1*	1	12	4.47	*MP2K1*	1	21	3.81
*ABL1*	1	22	5.37	*FGFR1*	1	16	6.31	*NOS1*	1	5	1.67
*ACE*	1	41	7.00	*FKB1A*	1	14	4.66	*NRAM*	1	15	5.58
*ACES*	1	42	6.41	*FNTA*	1	40	6.75	*PA2GA*	1	13	5.19
*ADA*	1	9	2.37	*FPPS*	1	10	3.96	*PARP1*	1	29	6.05
*ADA17*	1	35	8.24	*GCR*	1	21	4.23	*PDE5A*	1	18	3.99
*ADRB1*	1	21	3.71	*GLCM*	1	5	1.78	*PGH1*	1	9	2.71
*ADRB2*	1	19	3.89	*GRIA2*	1	8	2.55	*PGH2*	1	28	6.32
*AKT1*	1	33	8.15	*GRIK1*	1	10	1.90	*PLK1*	1	9	2.91
*AKT2*	1	10	3.28	*HDAC2*	1	25	7.29	*PNPH*	1	16	5.46
*ALDR*	1	10	2.23	*HDAC8*	1	33	9.11	*PPARA*	1	46	7.78
*ANDR*	1	22	4.70	*HIVINT*	1	7	2.20	*PPARD*	1	33	7.50
*AOFB*	1	4	1.57	*HIVPR*	1	50	8.74	*PPARG*	1	47	8.92
*BACE1*	1	23	3.81	*HIVRT*	1	18	4.21	*PRGR*	1	30	7.48
*BRAF*	1	11	3.04	*HMDH*	1	19	7.34	*PTN1*	1	9	2.19
*CAH2*	1	47	8.56	*HS90A*	1	8	3.04	*PUR2*	1	13	3.51
*CASP3*	1	13	3.55	*HXK4*	1	10	2.61	*PYGM*	1	5	2.11
*CDK2*	1	25	6.48	*IGF1R*	1	18	4.66	*PYRD*	1	13	4.38
*COMT*	1	5	2.69	*INHA*	1	5	2.16	*RENI*	1	14	4.38
*CP2C9*	1	5	1.80	*ITAL*	1	15	4.51	*ROCK1*	1	8	2.29
*CP3A4*	1	10	2.61	*JAK2*	1	6	1.94	*RXRA*	1	17	7.43
*CSF1R*	1	14	4.18	*KIF11*	1	10	3.66	*SAHH*	1	12	4.07
*CXCR4*	1	3	1.83	*KIT*	1	17	4.87	*SRC*	1	80	21.07
*DEF*	1	9	2.39	*KITH*	1	8	2.82	*TGFR1*	1	11	3.06
*DHI1*	1	19	4.16	*KPCB*	1	21	3.71	*THB*	1	22	6.10
*DPP4*	1	59	10.98	*LCK*	1	53	12.18	*THRB*	1	57	5.79
*DRD3*	1	75	12.35	*LKHA4*	1	15	4.46	*TRY1*	1	42	8.65
*DYR*	1	22	7.54	*MAPK2*	1	12	4.27	*TRYB1*	1	15	4.31
*EGFR*	1	56	9.08	*MCR*	1	6	2.31	*TYSY*	1	17	3.98
*ESR1*	1	67	21.07	*MET*	1	20	4.19	*UROK*	1	12	4.33
*ESR2*	1	63	20.80	*MK01*	1	6	2.24	*VGFR2*	1	48	10.42
*FA10*	1	29	5.51	*MK10*	1	7	1.85	*WEE1*	1	16	5.28
*FA7*	1	10	2.33	*MK14*	1	41	7.70	*XIAP*	1	25	6.32
*FABP4*	1	4	2.31	*MMP13*	1	39	8.14				

**Figure 5:**
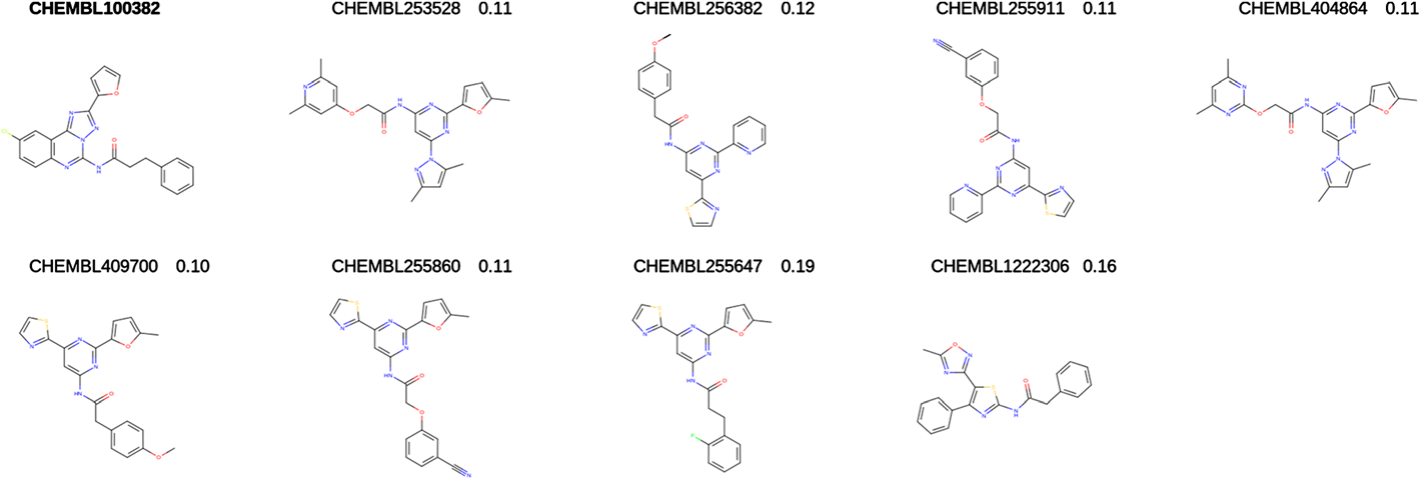
**Example of scaffold hopping with USRCAT.** The first structure in the top left corner is an active query molecule from DUD-E target *AA2AR*. The following compounds were only retrieved by USRCAT and not by topological similarity at an Enrichment Factor (EF) 0.25%. The Tanimoto similarity between the active query and these compounds is displayed to the right of the compound name.

Figure 5 shows an example of scaffold hopping with USRCAT. The first structure in the top left corner is an active query molecule from DUD-E target AA2AR and the following compounds were only retrieved by USRCAT and not by topological similarity at EF 0.25%. Moreover, the latter was only capable of retrieving one of those active hits at EF 1.0%.

## Conclusions

An extension to the USR algorithm that is capable of including atom type information was implemented and benchmarked in this analysis. The USRCAT extension was shown to improve the virtual screening performance of the original method significantly whilst preserving the ability to retrieve hits with very low structural similarity. It has to be stressed however that DUD-E was found to be not ideal to benchmark the virtual screening performance of global shape similarity algorithms such as USR and its variants due to the large variations in molecular size of the active ligands. Although USRCAT performed slightly better than ElectroShape in this benchmark, both methods appeared to reach similar limits of what is possible with the USR approach given the properties of DUD-E.

The USRCAT approach is simple and can easily be adapted to specific needs by selecting entirely different atom types to define subsets. The usage of “weights” for the pharmacophoric contributions in the similarity metric and not in the moments themselves allows users to change query parameters at runtime, unlike ElectroShape where the scaling factors are hard-coded in the calculated moments. The high speed and simplicity of the database implementation with possible usage of indexes makes the USRCAT method perfectly suitable for usage in combination with other virtual screening methods to identify as many starting points for lead generation as possible. Moreover, the algorithm can be implemented in any chemical informatics toolkit that supports SMARTS pattern matching. The source code of the USRCAT extension was released as a Python module under the MIT license and can be downloaded at http://hg.adrianschreyer.eu/usrcat.

## Methods

### Topological fingerprints

Four types of fingerprints from the OpenEye GraphSim TK (v. 2.0.2) were considered: Circular, Path, Tree and MACCS166. GraphSim TK fingerprints for DUD-E molecules were calculated with the help of *pgopeneye*, an OpenEye PostgreSQL cartridge (unpublished), which makes the required functions from several OpenEye C++ toolkits accessible as SQL functions. The functions to calculate similarity from GraphSim TK were re-implemented to take advantage of the SSE4 POPCNT instructions found in modern CPUs that can significantly increase performance [25]. In addition, the data type from the cartridge used to represent OpenEye fingerprints in the database also supports Generalized Search Tree (GIST) indexes to speed-up the use of queries. All fingerprints were created with their default atom and bond types and a bit length of 2048.

### Calculating ultrafast shape recognition moments with CREDO atom types

Ultrafast Shape Recognition with CREDO Atom Types (USRCAT) extends the USR method to also include pharmacophore information. This was implemented by calculating additional USR moments for specific subsets of a molecule’s atoms, with the subsets in this work being defined as either hydrophobic, aromatic, hydrogen bond donor or acceptor atoms. Subset atoms were identified with the help of SMARTS patterns that are used for atom typing in the CREDO database. Unlike UFSRAT, the four reference points derived from all atom coordinates are used to calculate the distributions for the subset moments to improve screening performance (and *not* calculated for each subset). The three moments that were calculated to describe each distribution are the mean, the standard deviation (square root of variance) and the cube root of the distribution’s skewness. The result is a USRCAT vector with 60 elements (5x12) with the first 12 being identical to traditional USR moments. In the case of an empty subset, for examples if no hydrogen bond donors are found, then the corresponding elements in the moment vector will be set to zero. Similarity between two USRCAT vectors *i* and *j* is calculated in the same manner as USR with a variant of the Manhattan distance with the exception that each set of 12 moments can be scaled to give a higher (or lower) weight to a certain pharmacophore (equation 1). The five scaling factors are *ow* (all atoms), *hw* (hydrophobes), *rw* (aromatic atoms), *aw* (acceptors) and *dw* (donors).

(1) $$\begin{array}{l} S\left(i,j,ow,hw,rw,aw,dw\right)\\ \;=\frac{1}{\begin{array}{l} 1.0+ow\left(\frac{1}{12}\sum_{l=1}^{12}\left|M_{l}^{i}-M_{l}^{j}\right|\right)+\\ hw\left(\frac{1}{12}\sum_{l=13}^{24}\left|M_{l}^{i}-M_{l}^{j}\right|\right)+\\ rw\left(\frac{1}{12}\sum_{l=25}^{36}\left|M_{l}^{i}-M_{l}^{j}\right|\right)+\\ aw\left(\frac{1}{12}\sum_{l=37}^{48}\left|M_{l}^{i}-M_{l}^{j}\right|\right)+\\ dw\left(\frac{1}{12}\sum_{l=49}^{60}\left|M_{l}^{i}-M_{l}^{j}\right|\right) \end{array}}\in \left[0,1\right] \end{array}$$

The USRCAT method was implemented in the Python programming language with the help of the NumPy and SciPy modules as well as the Python wrappers of OpenEye’s OEChem TK. In terms of performance, the software generates 2,100 USRCAT moments/s on average using a single thread. The Python module also supports RDKit as a backend and its source code is available at http://hg.adrianschreyer.eu/usrcat under the MIT License. The repository also contains the used SMARTS patterns.

### Implementation of USRCAT in the PostgreSQL relational database management system

All database functions have been executed on a dedicated server with two Intel Xeon X5650 processors, 48GB RAM and 2TB hard drives in RAID10 configuration. PostgreSQL version 9.1 was used as relational database management system (RDBMS). Methods to generate USRCAT moment vectors and to calculate similarities between them were implemented natively in PostgreSQL. This has the advantage that additional constraints of arbitrary complexity can be easily added to a query, such as physicochemical properties of molecules, to consider affinity data from the ChEMBL database or even to exclude ligands that bind to a specific target. USRCAT moments are stored in PostgreSQL as a domain of type double precision array (arrayxd) with the constraints of only a single dimension and no NULL values. The domain was implemented with the help of *pgeigen*, an extension that uses the Eigen C++ template library [26] to add numerical data types and functions to PostgreSQL. The source code of *pgeigen* is also released under the MIT license and can be downloaded from http://hg.adrianschreyer.eu/pgeigen.

The USRCAT database implementation exploits the cube data type that is part of the PostgreSQL contrib module [27]. The cube extension can be used to store multi-dimensional hypercubes and perform calculations on them. More importantly however, the cube data type has GIST index support to find the intersections between cubes. The USRCAT implementation uses the first 12 moments (identical to traditional USR) to create a 12-dimensional cube in a second column. Similar molecules can be found in a bounding box that is created by enlarging the reference cube by a given radius across all twelve dimensions. This operation is supported by the GIST index operator class for cube values and therefore avoids linear scans of all USRCAT moments. The performance was further improved by clustering the index on the column that contains the first 12 USR moments as cube data type with the CLUSTER command. Clustering simply means that the table data on disk is physically reordered based on the index information, i.e. similar items are more likely to be on the same table page (as the query) thereby saving unnecessary disk accesses. The code of an example query demonstrating the SQL implementation of USRCAT is available online at https://gist.github.com/3690246.

### Calculating ElectroShape moments with partial charges and lipophilicity

ElectroShape is a variant of the USR method that adds electrostatic and lipophilic information through additional dimensions and centroids [11,12]. The ElectroShape method was also implemented as a Python module since there is no reference implementation provided by the authors. The method was implemented according to the authors’ descriptions with the following exceptions: atomic partial charges and lipophilicities were calculated with OEChem instead of MOE ( http://www.chemcomp.com). Partial charges were assigned with the help of the OEMMFF94PartialCharges function since MMFF94 achieved the best results in the original ElectroShape benchmark. Lipophilicity was calculated with OEGetXLogP from OpenEye’s MolProp toolkit because alogP was not available. The same conformers were used for all methods described in this work. Atomic partial charges and lipophilicities have their own units while the spatial dimensions (first three) are given in Ångstroms. The authors therefore used scaling factors for conversion of the new dimensions and the combination of 25Å for partial charges and 5Å for each lipophilicity unit resulted in the highest enrichment factor in their benchmark. Hence the same values were used in this ElectroShape implementation as default. The source code of the ElectroShape Python module is available at http://hg.adrianschreyer.eu/electroshape.

### Virtual screening of chemical components found in the protein data bank

A relational table containing USRCAT moments was also created for chemical components found in the PDB (part of the CREDO database). OpenEye’s Omega TK (v. 2.4.6) was used to generate up to 200 conformers with default settings for each structure, leading to a total of 1,501,895 conformers for the 14,856 chemical components currently stored in CREDO (August 2012). USRCAT moments were generated for all conformers and stored in the database and afterwards indexed and clustered as described above.

### Virtual screening using the DUD-E database

Virtual screens of the DUD-E database were performed with topological fingerprints and USRCAT moments. Topological fingerprints were created for all 1,431,121 molecules in DUD-E – to compare the performance of the shape-based classic USR and the new USRCAT algorithm with a well-established virtual screening method but also to be able to analyse the scaffold hopping potential by calculating the topological similarity between the actives used as queries and those that were retrieved. DUD-E target *AMPC* was ignored because the selected actives did not appear to have been drawn from ChEMBL (no ChEMBL identifiers). The USRCAT algorithm requires three-dimensional coordinates in order to produce meaningful statistical moments that can be used to compare the shape of molecules. DUD-E already provides a single conformer with three-dimensional coordinates for all actives and decoys. However, up to 20 new conformers were generated for the actives and decoys of all targets in DUD-E since a molecule can easily possess a range of reasonable conformations that could significantly affect screening performance. OpenEye’s Omega TK (v. 2.4.6) was used for this purpose with default settings. As a result, 27,069,902 conformers were generated for all molecules in DUD-E, leading to an average of 19 per molecule. USRCAT moments were subsequently calculated and stored in a partitioned database table (by target). Moments and similarity values for the traditional USR method were not calculated separately but simply obtained through usage of USRCAT. It will produce the same results if the all atoms weight (*ow*) is set to 1.0 and all others to 0.0 because the first 12 moments are identical. Only the Lowest Energy Conformer (LEC) of an active molecule was used as the reference geometry to screen all conformers of the compounds set (active and decoys) of a particular target.

The virtual screening benchmarks for topological fingerprints and USRCAT are carried out as follows. For each target in DUD-E, all actives are fetched. Each active is used as a reference for searching against the whole dataset (actives plus decoys) for this target, excluding the query active to avoid an artificial boost in performance. The 1% top-ranking hits are retrieved and the Enrichment Factor (EF) calculated at the 1%, 0.5%, 0.25% levels (equation 2). The enrichment factors were then averaged for all actives of a target that were used as queries and finally averaged to provide the average performance of the method. The EF is the proportion of true positives (the actives) in the set of compounds retrieved over the proportion of true positives that would be obtained by screening the whole database [5].

(2) $$EF_{ij,x\%}=\frac{a_{ij,x\%}/d_{ij,x\%}}{A_{i}/D_{i}}$$

*a*_*ij*,*x%*_ is the number of actives retrieved at the top *x*% of the query generated by the *j*th active template from the *i*th target, *d*_*ij*,*x%*_ the number of retrieved decoys, *A*_*i*_ the total number of ligands and *D*_*i*_ the total number of Decoys for the *i*th target.

## Competing interests

The author(s) declare that they have no competing interests.

## Authors’ contributions

AMS designed and implemented the code, carried out the benchmarks and drafted the manuscript. TB provided feedback and proofread the manuscript. Both authors read and approved the final manuscript.

## Supplementary Material

Additional file 1 Comparison of enrichment factors for USR and USRCAT across all targets. Click here for file
